# In vivo MRI evaluation of early postnatal development in normal and impaired rat eyes

**DOI:** 10.1038/s41598-021-93991-2

**Published:** 2021-07-30

**Authors:** Jeannie M. Au, Swarupa Kancherla, Malack Hamade, Monica Mendoza, Kevin C. Chan

**Affiliations:** 1 Department of Ophthalmology, NYU Grossman School of Medicine, NYU Langone Health, New York University, New York, NY USA; 2 Department of Ophthalmology, University of Pittsburgh, Pittsburgh, PA USA; 3 Department of Radiology, NYU Grossman School of Medicine, NYU Langone Health, New York University, New York, NY USA; 4 Neuroscience Institute, NYU Grossman School of Medicine, NYU Langone Health, New York University, New York, NY USA; 5 Center for Neural Science, College of Arts and Science, New York University, New York, NY USA; 6 Department of Biomedical Engineering, Tandon School of Engineering, New York University, New York, NY USA; 7 Department of Electrical and Electronic Engineering, The University of Hong Kong, Pokfulam, Hong Kong SAR China

**Keywords:** Visual system, Magnetic resonance imaging, Eye abnormalities

## Abstract

This study employed in vivo 7-T magnetic resonance imaging (MRI) to evaluate the postnatal ocular growth patterns under normal development or neonatal impairments in Sprague–Dawley rats. Using T2-weighted imaging on healthy rats from postnatal day (P) 1 (newborn) to P60 (adult), the volumes of the anterior chamber and posterior chamber (ACPC), lens, and vitreous humor increased logistically with ACPC expanding by 33-fold and the others by fivefold. Intravitreal potassium dichromate injection at P1, P7, and P14 led to T1-weighted signal enhancement in the developing retina by 188–289%. Upon unilateral hypoxic-ischemic encephalopathy at P7, monocular deprivation at P15, and monocular enucleation at P1, T2-weighted imaging of the adult rats showed decreased ocular volumes to different extents. In summary, in vivo high-field MRI allows for non-invasive evaluation of early postnatal development in the normal and impaired rat eyes. Chromium-enhanced MRI appeared effective in examining the developing retina before natural eyelid opening at P14 with relevance to lipid metabolism. The reduced ocular volumes upon neonatal visual impairments provided evidence to the emerging problems of why some impaired visual outcomes cannot be solely predicted by neurological assessments and suggested the need to look into both the eye and the brain under such conditions.

## Introduction

Vision involves the perception of light through the eye and the brain. Upon entry to the eye, light rays refract at the boundaries between the cornea, anterior chamber, posterior chamber, lens, and vitreous humor before focusing on the retina for visual processing in the neural pathways. Comprehending how each structure develops in normal and impaired eyes is pertinent to improving the maintenance of ocular integrity and visual perception when visual development is disturbed.

Research on eye development often requires post-mortem tissue processing or tissue sectioning, which can affect the true morphology of the eye^1^. In contrast, in vivo imaging allows non-destructive and longitudinal monitoring of ocular growth in the same subjects. It can also reveal morphological changes more accurately under physiological conditions while minimizing sampling errors from biovariability between groups^2^. Among the in vivo imaging techniques, optical imaging offers high resolution down to cellular levels^3^. However, optical opacities from the iris and sclera render whole-eye assessment difficult with the need to correct for optical distortions produced by ocular boundaries^4,5^. Furthermore, optical imaging remains limited for ocular growth studies in utero, before natural eyelid opening, or under impaired conditions when the eye is not optically accessible, such as cloudiness in the cornea and lens^6^. Ultrasound has deeper penetrating abilities, though the field of view, resolution, and sensitivity remain challenging for whole-globe imaging^7^.

In contrast, magnetic resonance imaging (MRI) has no depth limitation and allows for non-invasive assessments of the whole eye^8–11^. To date, MRI assessments of early ocular development remain sparse^12–14^. The aim of this study is to use MRI to evaluate the normal growth of the rat eye from birth to maturity, followed by the examinations of how neonatal impairments including hypoxic-ischemic encephalopathy (HIE), monocular deprivation (MD), and monocular enucleation (ME) alter the development of the eye. For normal postnatal ocular growth, we used high-field anatomical T2-weighted imaging to evaluate early development in the healthy rat eyes before and after natural eyelid opening at around postnatal day (P) 14, which is comparable to human eyelid opening at 25.5 to 26.5 gestational weeks^15^. We hypothesized that ocular structures including the anterior chamber and posterior chamber (ACPC), lens, and vitreous humor (VH) follow a logistic growth pattern during early postnatal normal development (ND) in the healthy rats. Apart from T2-weighted imaging, contrast-enhanced MRI was performed using the positive T1 contrast agent potassium dichromate^16,17^ to assess normal postnatal growth in the developing rat retina before natural eyelid opening.

For neonatal impairments, neonatal HIE is one of the leading causes of infant death, with 25% of pediatric survivors living with neurodevelopmental impairments including poor visual outcomes^18^. Although HIE possesses a profound degenerative effect on the neuronal cells and white matter^19,20^, neuroimaging alone appears inadequate in assessing the extent of visual impairment from HIE^21^. Here, we hypothesized that unilateral HIE results in a significant volumetric reduction in the ACPC, lens, and VH bilaterally coinciding with brain injuries.

MD is a model for amblyopia in which input from one eye is preferred over the remaining eye, creating a mismatch between the views from each eye^22^. On the other hand, ME is commonly performed in pediatric survivors of retinoblastoma^23^. To date, it is unclear how MD and ME may affect the development of the remaining eyes. We hypothesized that MD and ME can slow down the growth of the ACPC, lens, and VH in the surviving eyes. While MRI studies of human ocular development are generally constrained to limited time points^24^, the results of our in vivo preclinical experiments can be important in not only modeling postnatal ocular development longitudinally, but also offering insights into the ocular involvements in different types of early visual impairments for guiding intervention.

## Results

### Normal postnatal development of ocular structures

**Figure 1:**
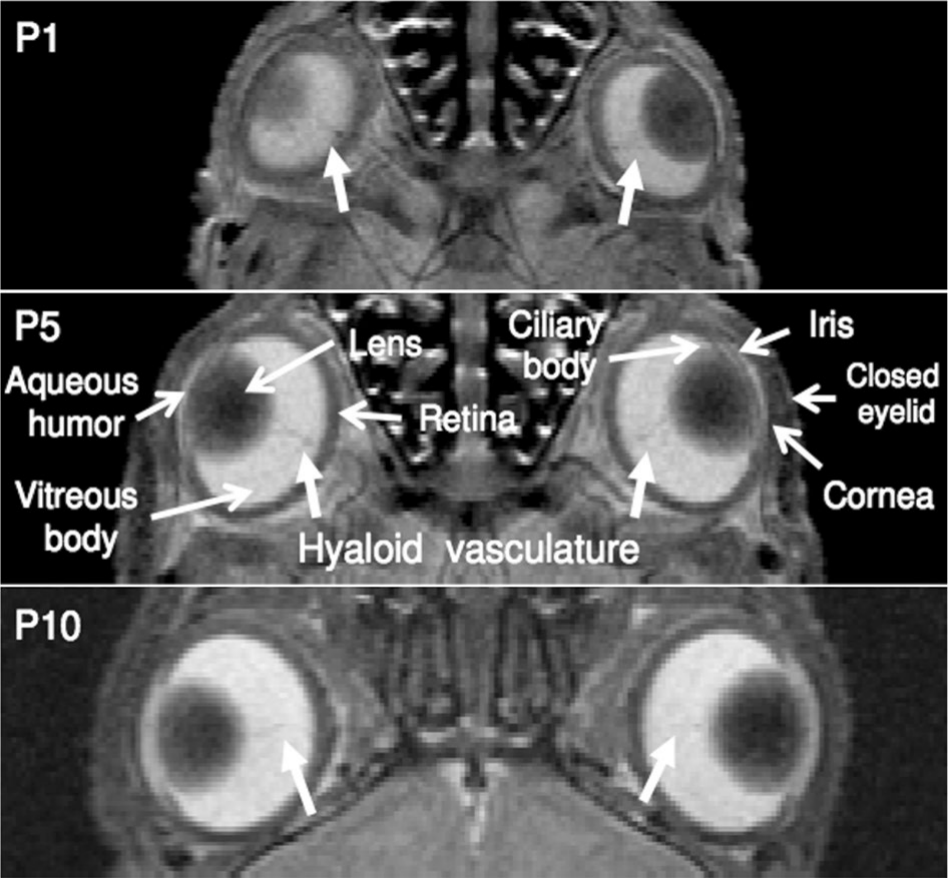
T2-weighted imaging of early postnatal ocular development before natural eyelid opening at postnatal day (P) 14 in Group 1. The lens, vitreous humor, and retina are discernable at P1, P5, and P10, whereas the anterior chamber and posterior chamber appear underdeveloped with the hyperintense aqueous humor gradually forming behind the fused eyelids (open arrows). Note also the hyaloid vasculature in the vitreous at all 3 ages (closed arrows).

**Figure 2:**
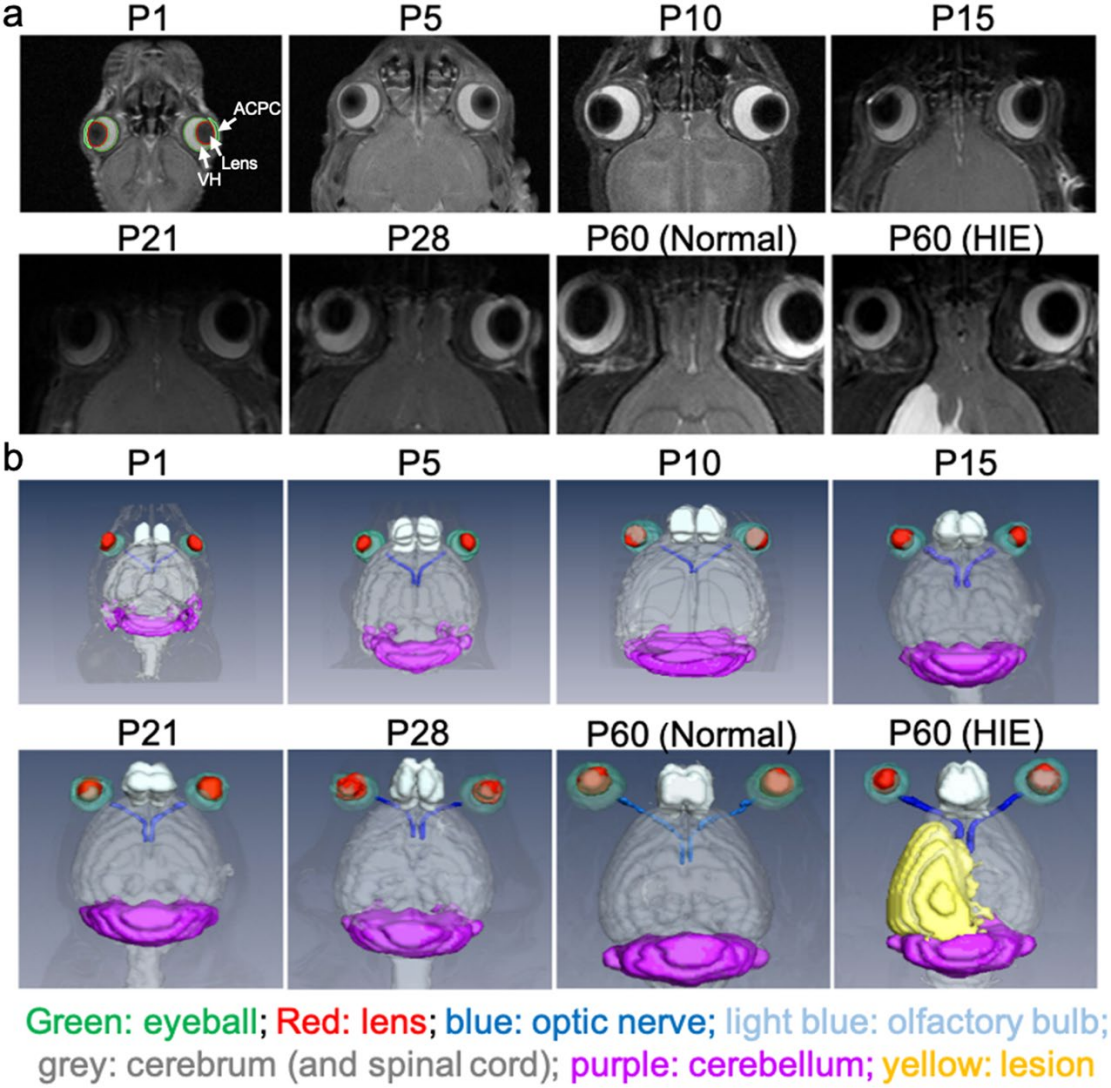
In vivo magnetic resonance microscopy of normal postnatal ocular development from postnatal day (P) 1 to 60 in Group 1, and at P60 after unilateral hypoxic-ischemic encephalopathy (HIE) at P7 in Group 3. (**a**) T2-weighted imaging at the level of the eyes. Manual delineation of the anterior chamber and posterior chamber (ACPC), lens, and vitreous humor (VH) was illustrated by overlaying their regions of interest on the image at P1; (**b**) Volume-rendered and color-labeled eye and brain structures of the same animals. Note the large lesion in the left hemisphere of the HIE group at P60. Note also the generally comparable bilateral growth in normally developing eyes, and the smaller left eye in the HIE group by P60.

**Figure 3:**
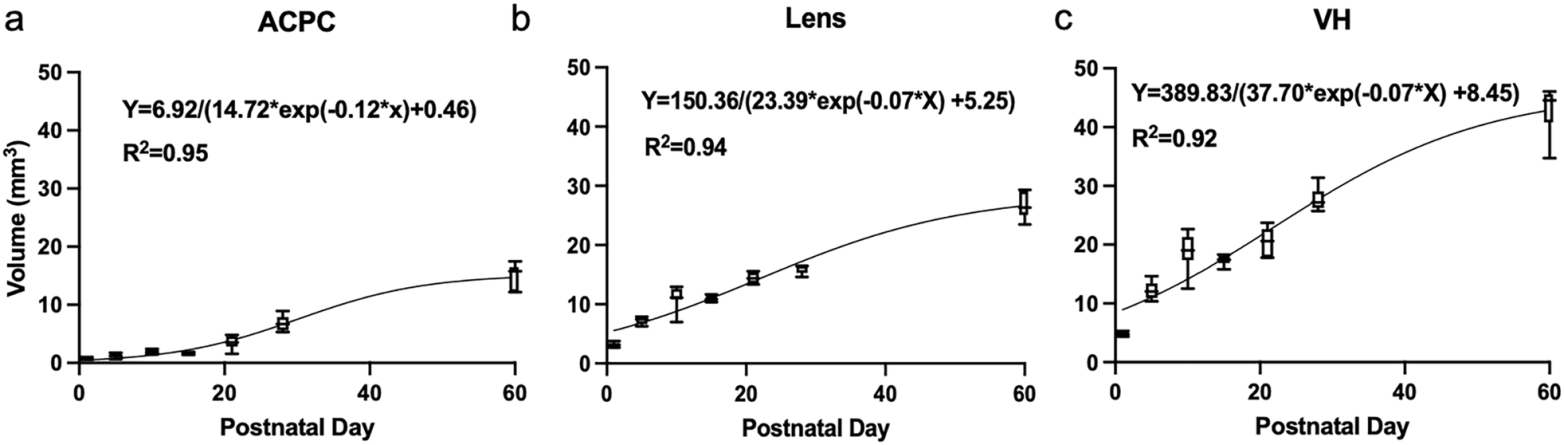
Quantitative evaluation of normal ocular growth in the rat (**a**) anterior chamber/posterior chamber (ACPC), (**b**) lens, and (**c**) vitreous humor (VH) from postnatal day 1 to 60 in Group 1. In descending order, values in box and whisker plots represent: 95 percentile, third quartile, median, first quartile, and 5 percentile. Using non-linear regression modeling, ACPC, lens, and VH followed the logistic growth curves as indicated in each panel, where Y denotes volume in mm^3^ and X denotes postnatal day.

To examine the normal postnatal ocular development, T2-weighted imaging was acquired in a 7-T MRI scanner on seven ND Sprague–Dawley rats (Group 1) at 7 time points from P1 (newborn) to P60 (adult). Before natural eyelid opening at around P14, the lens, VH, retina, and hyaloid vessels were discernable in T2-weighted imaging. In contrast, the ACPC appeared less discernable and underdeveloped with the hyperintense aqueous humor gradually forming behind the fused eyelids from P1 to P10 (Fig. 1). After natural eyelid opening, these ocular structures continued to grow toward adulthood at P60 (Fig. 2). Quantitatively, the ACPC, lens, and VH followed the logistic growth curves with good fittings of R^2^ = 0.92 or above (Fig. 3). In terms of individual parameters derived from the logistic growth model, the ACPC was the smallest in size near birth followed by the lens (11.5 times larger than ACPC) and VH (18.5 times larger than ACPC), whereas at the maximum volume, the lens was 1.9 times larger than ACPC and the VH was 3.0 times larger than ACPC (Table 1). The ACPC grew at the fastest rate postnatally with a 33-fold expansion from newborn to adult stages as compared to approximately fivefold increases in both the lens and VH.

**Table 1:** Parameters of the logistic growth model in the anterior chamber/posterior chamber (ACPC), lens, and vitreous humor (VH) of normally developing rats from postnatal day 1 to 60.

Structure	Y_0_ (mm^3^)	Y_M_ (mm^3^)	k (day^−1^)
ACPC	0.46 (0.22 to 0.79)	15.18 (14.16 to 16.53)	0.12 (0.09 to 0.15)
Lens	5.25 (4.44 to 6.10)	28.64 (26.41 to 31.73)	0.07 (0.06 to 0.08)
VH	8.45 (6.98 to 10.01)	46.15 (42.17 to 51.96)	0.07 (0.05 to 0.08)

The logistic growth curve follows the equation Y = Y_M_ * Y_0_/[(Y_M_ − Y_0_) * exp(− k * X) + Y_0_], where Y is volume in mm^3^, X is postnatal days, Y_0_ is the fitted volume at birth in mm^3^; Y_M_ is the maximum volume in mm^3^, and k is the rate constant in inverse unit of X (day^-1^). Data are represented as “Best fit (95% confidence interval)”.

### Chromium-enhanced MRI of the developing retina

**Figure 4:**
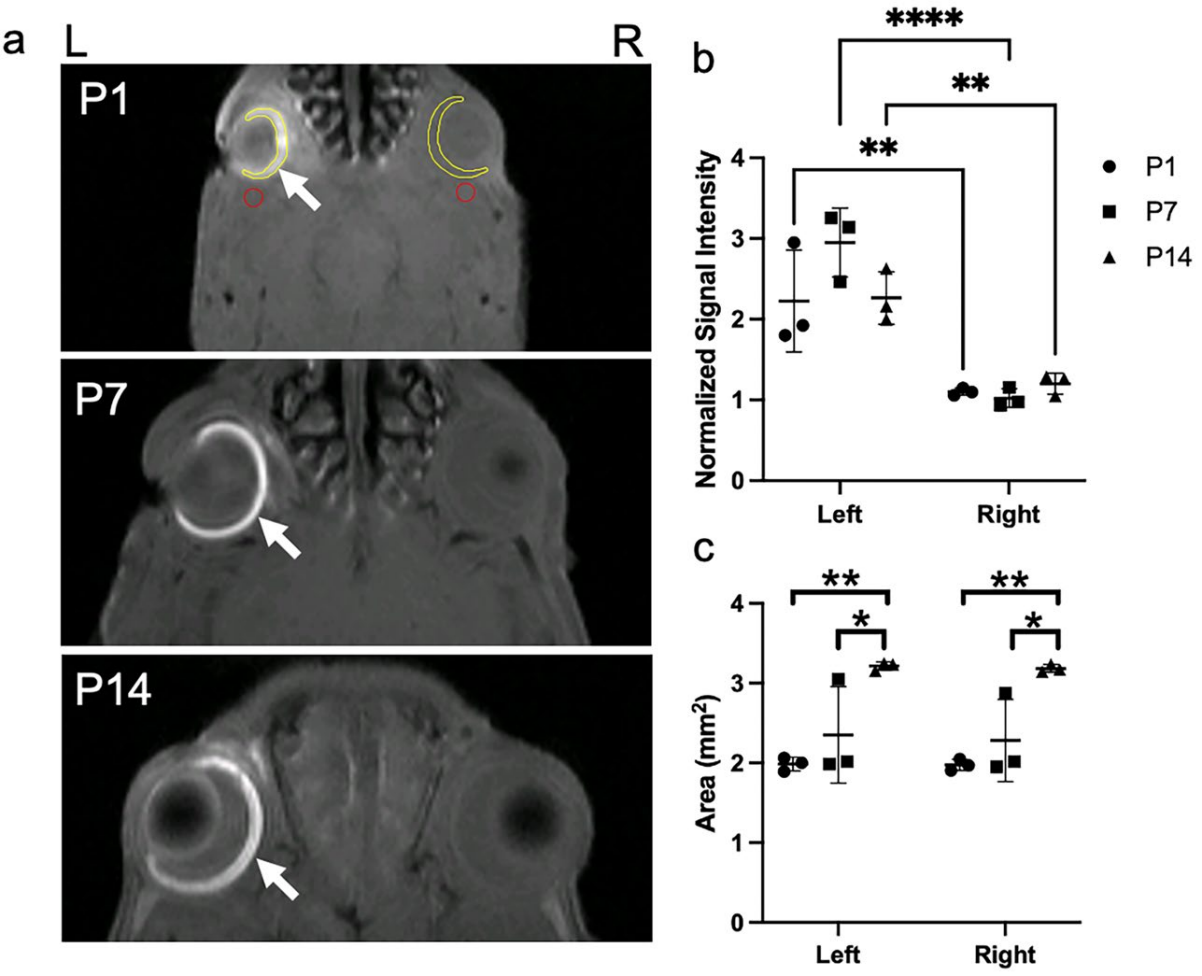
(**a**) T1-weighted chromium-enhanced MRI of the developing retina at one day after unilateral intravitreal potassium dichromate injection at postnatal days (P) 1, 7, and 14 in Group 2. Note the signal enhancement in the left injected retina at all ages (arrows). Regions of interest (ROI) were manually delineated in the retina (yellow) and the surrounding muscles (red) of both hemispheres for quantitative comparisons in (**b**) and (**c**); (**b**) Normalized signal intensity of the retina at P1, P7, and P14 in the left and right eyes. Normalization was performed by dividing the signal intensity of the retina to that of the surrounding muscles; (**c**) Area of the retina at P1, P7, and P14 in the left and right eyes. Note the significant increases in retinal areas at P14 relative to P1 and P7. Data are presented as mean ± standard deviation. *p < 0.05, **p < 0.01, ***p < 0.001, ****p < 0.0001 for post-hoc Sidak multiple comparisons correction tests.

To demonstrate the compatibility of chromium (Cr)-enhanced MRI in studying the developing retina, T1-weighted imaging was performed on nine neonatal ND rats (Group 2) after unilateral intravitreal Cr injection (Fig. 4a). Quantitatively, the signal intensity in the left Cr-injected retina was enhanced to 202%, 289%, and 188% of the signal intensity in the right non-injected retina one day after Cr injection at P1, P7, and P14 respectively (Fig. 4b). The retina appeared to grow at a faster rate between P7 and P14 than between P1 and P7 (Fig. 4c). No apparent size difference was observed between the left and right retinas at each time point.

### Neonatal hypoxic-ischemic encephalopathy

**Figure 5:**
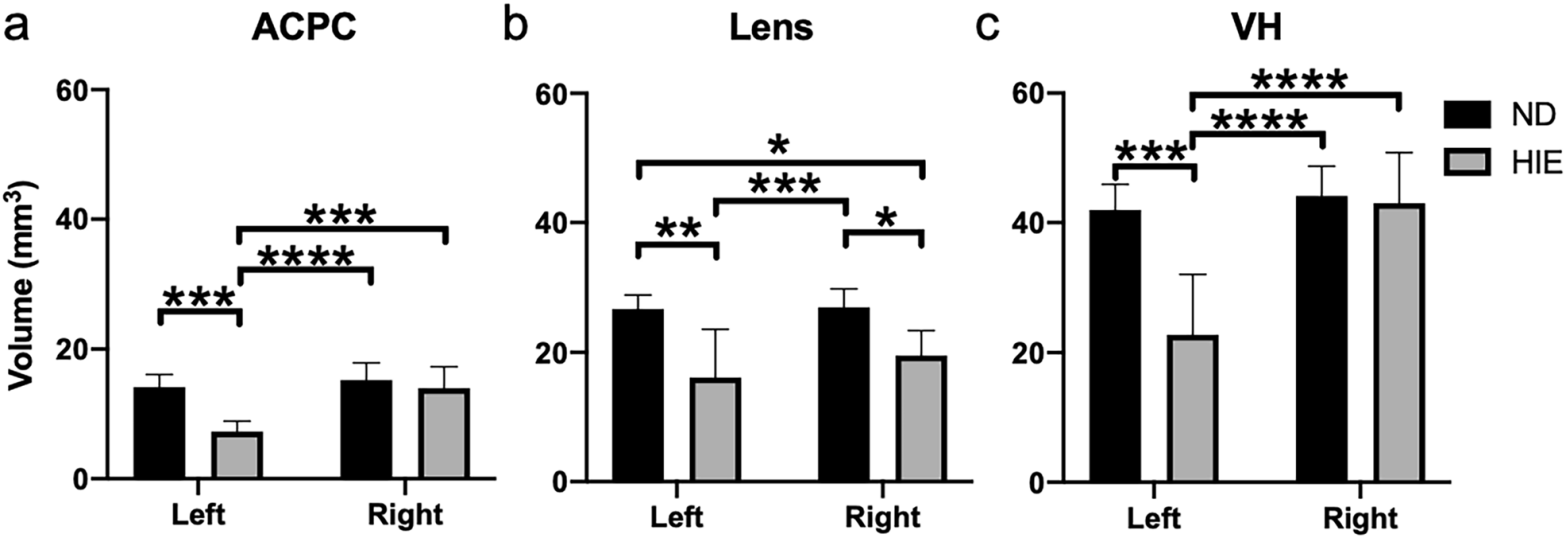
Quantitative evaluation of the volumes of the (**a**) anterior chamber and posterior chamber (ACPC), (**b**) lens, and (**c**) vitreous humor (VH) in unilateral hypoxic-ischemic encephalography (HIE) (Group 3) and normally developing (ND) rats (Group 1) at postnatal day (P) 60. Data are presented as mean ± standard deviation. *p < 0.05, **p < 0.01, ***p < 0.001, ****p < 0.0001 for post-hoc Sidak multiple comparisons correction tests.

To evaluate how neonatal impairments can alter ocular development, we examined 7 adult rats (Group 3) that had undergone ligation of the left common carotid artery followed by 2-h hypoxia at P7. These HIE rats are all presented with a T2-weighted hyperintense lesion at P60 covering the majority of the left hemisphere (Fig. 2). Quantitatively, at P60, the left eye of the HIE rats had about one half less ACPC volume relative to the contralateral right eye of the same rats, or either eye of the ND rats in Group 1 (Fig. 5a). The lens of the left eye of the HIE rats was indifferent from that of the contralateral right eye in volume, but was smaller than the lens of either eye in the ND rats (Fig. 5b). Furthermore, the left VH of the HIE rats was smaller than the contralateral right eye as well as the ND eyes of either side (Fig. 5c). In the right eye of the HIE rats, the lens was smaller in volume compared to the ND eyes of either side, whereas the right ACPC and VH of the HIE rats were indifferent in volume from those of the ND rats (Fig. 5a–c).

### Monocular deprivation and monocular enucleation

**Figure 6:**
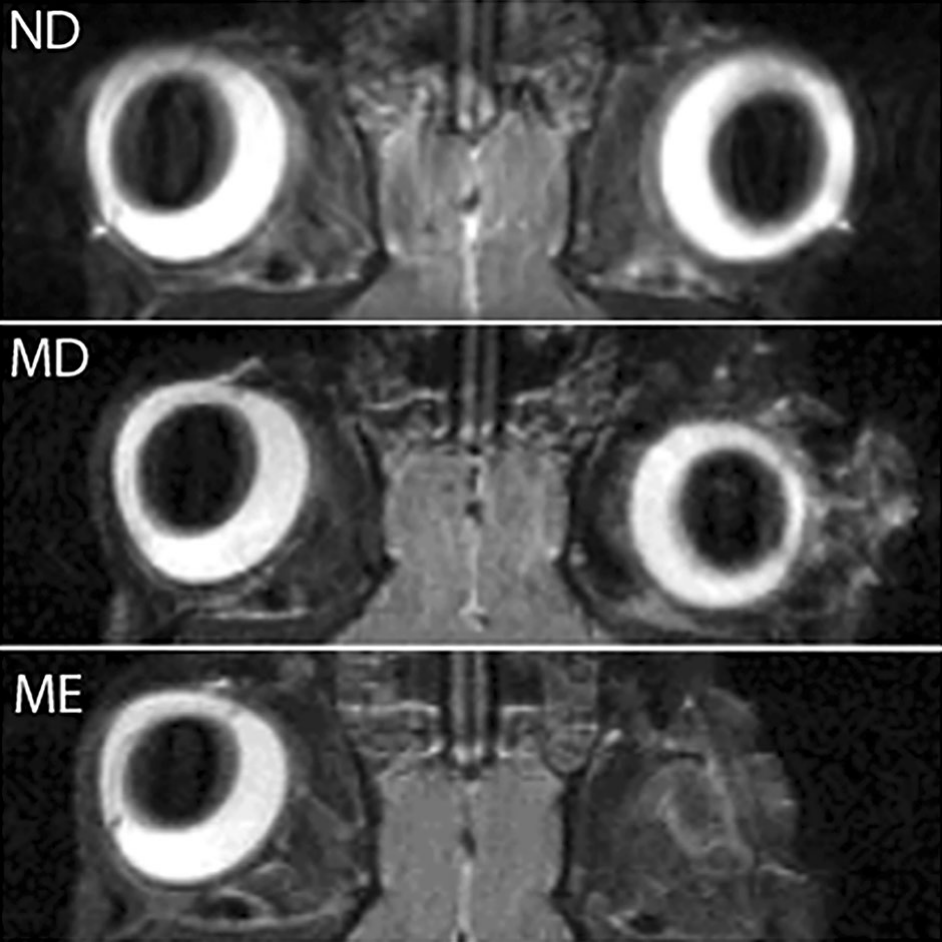
T2-weighted imaging of ocular components at postnatal day (P) 45 during normal development (ND, top) in Group 6, after monocular deprivation (MD, middle) to the right eye at P15 in Group 4, or after monocular enucleation (ME, bottom) to the right eye at P1 in Group 5. Note the absence of the right eye in the ME group, and the smaller right eye in the MD group.

**Figure 7:**
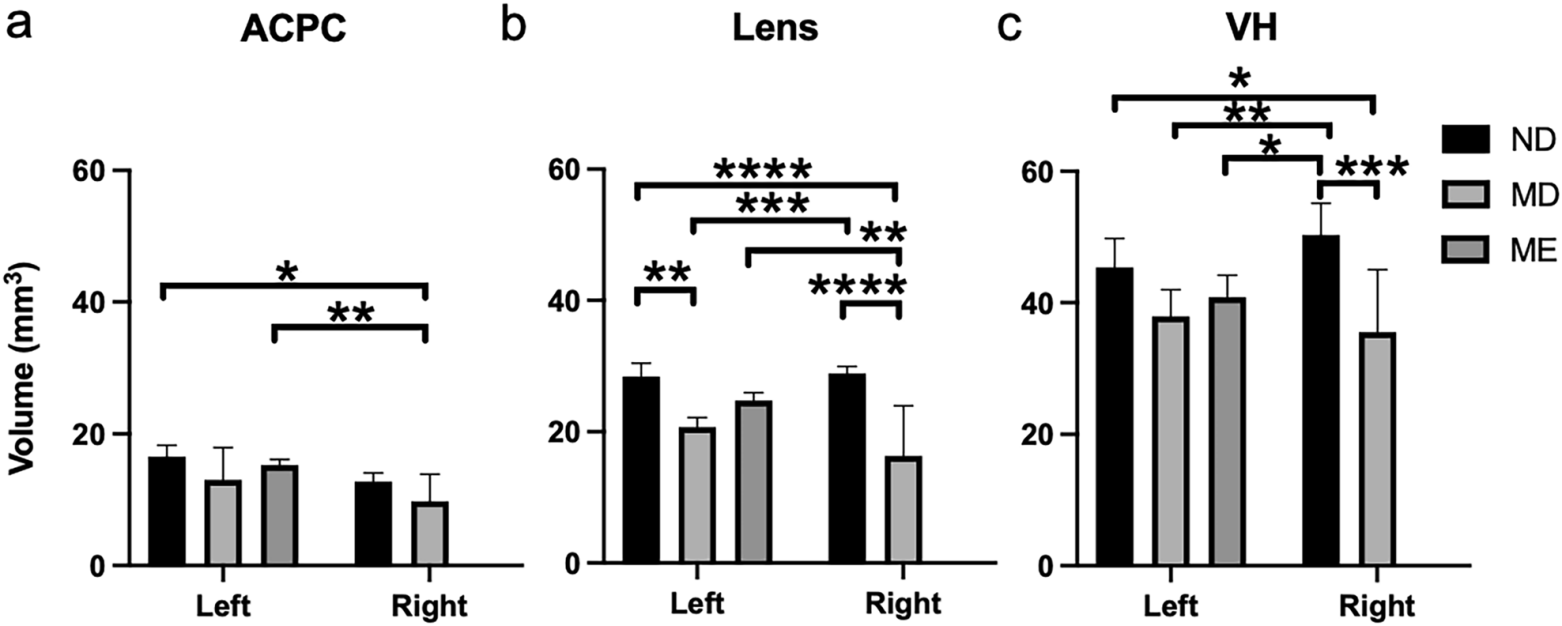
Quantitative evaluation of the (**a**) anterior chamber and posterior chamber (ACPC), (**b**) lens, and (**c**) vitreous humor (VH) volumes in monocularly deprived (MD, Group 4), monocularly enucleated (ME, Group 5), and normally developing (ND) rats (Group 6) at postnatal day (P) 45. Data are presented as mean ± standard deviation. *p < 0.05, **p < 0.01, ***p < 0.001, ****p < 0.0001 for post-hoc Sidak multiple comparisons correction tests.

Apart from unilateral neonatal HIE, T2-weighted imaging was acquired at P45 on six MD rats (Group 4) after suturing the eyelid of the right eye at P15, five ME rats (Group 5) after enucleating the right eye at P1, and eight separate ND rats (Group 6). T2-weighted images indicated the absence of the eye on the enucleated side of the ME rats, as well as the visibly smaller right eye of the MD rats relative to the right eye of the ND rats (Fig. 6). Quantitatively, the right eye of the MD rats had smaller ACPC than the left eye of the ME and ND rats (Fig. 7a). For the lens, the right eye of the MD rats was smaller than the left eye of the ME rats and either eye of the ND rats, whereas the left eye of the MD rats was smaller than either eye of the ND rats (Fig. 7b). For the VH, the right eye of the MD rats was smaller than either eye of the ND rats, whereas the left eye of the MD rats was smaller than the right eye of the ND rats. The left eye of the ME rats also had smaller VH than the right eye of the ND rats (Fig. 7c).

## Discussion

This study shows the feasibility of high-field MRI for non-invasive detection of early postnatal ocular growth before natural eyelid opening, enabling in vivo assessment of initial ocular development in rats and other species. Before P14, rat eyelids grow over the cornea acting as a substrate^25^, whereas the VH and retinal surfaces are covered by the hyaloid vasculature for temporary circulation in both fetal and neonatal eyes^26^. Using T2-weighted imaging, the development of the hyperintense fluidic chambers and isointense ocular structures can be assessed non-destructively behind the fused eyelids of the same animals. In addition, the hypointense hyaloid vessels can also be detected until they regress and are taken over by the retinal blood vessels^27^. The same ocular structures can also be continuously monitored toward adulthood as the eyelid margin differentiates and opens allowing substantial visual input for guiding late postnatal growth^25^.

During normal rat eye development, the lens and VH begin maturation prior to birth, while ACPC is underdeveloped and barely visible at birth. The rat ACPC grows at a higher rate than the lens and VH after birth similar to young rhesus monkeys^28^ and humans^29^. This may be explained by the continuous cellular necrosis and macrophage formation that open up the intertrabecular spaces within the same postnatal period^30^. The faster channel formation in the rat angle from P5 to P60 is marked by a greater number of dying cells compared to earlier postnatal days^31^. By P5, the anterior chamber angle development of rats matches that of humans in the 15th gestational week in electron microscopy experiments^32^, which represents a viable model for understanding early human ACPC development. Rat lens growth slows down postnatally likely because of the slowed cell division and lens fiber differentiation induced by fibroblast growth factors^33^. In addition, the tunica vasculosa lentis regresses toward young adult stage and causes the lens to lower its overall metabolism. This requires the newly avascular lens to receive nutrients from the aqueous humor and potentially explains the slowed growth of the ACPC around the same period^34,35^. The VH supplies nutrients to the lens and gives support to the retina, orchestrating overall eye growth^36^. The VH has the largest volume at birth among all measured ocular structures and quintuples until its maximum volume. The postnatal growth of VH is slower than ACPC possibly because of the presence of hyalocytes that originate from the regression of the hyaloid vasculature^37^. Hyalocytes are characterized as tissue macrophages and have an inhibitory effect on VH growth^38,39^ but its role has not been conclusive^40^. Our in vivo imaging model system in this study may offer a non-invasive tool for longitudinal investigation into the role of hyaloid regression in VH growth in future studies.

Chromium-enhanced MRI may reflect lipid metabolism in the biological tissues due to their lipid peroxidation with the paramagnetic chromium contrast^17^. In adult rats, lipid content is the highest in the outer retina and is concentrated in the cone and rod outer segments^41,42^. In neonatal rodents, the retina is largely composed of neuroblastic cells, some of which differentiate into photoreceptor cells as early as at birth and increase their numbers until P14^43^. The chromium enhancement observed in the retina of ND rats after intravitreal injection at P1, P7 or P14 suggest viable detection of lipid contents from the rudimentary rods and cones in the developing retina before natural eyelid opening^25^. The indifferent retinal area between the injected left eye and non-injected right eye indicated no apparent effect of chromium on retinal growth in size within the experimental periods, whereas the more rapid retinal growth observed at P7 to P14 relative to P1 to P7 concurred with the patterns of outer retinal network development^25,27,44^. In neonates, the amount of simple lipids in the rodent retina decreases from birth while the amount of phospholipids, diacylglycerols, and triacylglycerols increases throughout the first 2 postnatal weeks^45^. Although we did not observe significant difference in signal enhancement across P1, P7, and P14 with the current small sample size, future studies may explore the contributions of different retinal lipids to the chromium enhancement in order to differentiate the developmental patterns in retinal metabolism.

Pediatric survivors of neonatal HIE often live with poor visual outcomes^18^. While the majority of previous studies have delved into such visual impairments by focusing on the neurological effects of HIE^46,47^, brain imaging alone appears inadequate in predicting the visual outcomes of HIE^21,48^. The impact of postnatal eye development depends on various factors including the extents of prematurity, oxygen, inflammation, and nutrition^49^. Recent findings suggest that neonatal HIE results in profound alterations in the retinal vasculature^50^. Global hypoxia can also suppress the formation of growth factors and the proliferation of epithelial cells in the lens, leading to reduced lens volume in both eyes^51^. In this study, our observations of reduced ACPC, lens, and VH volumes in the HIE rats support the notion that eye injury may contribute to visual impairments along with brain injury in the asphyxiated newborns. This warrants further research to better understand the disease processes of not only the brain but also the eye in order to develop therapeutic strategies to protect both for infants exposed to HIE. For example, longitudinal assessments of both the eye and the brain may help dissociate visual impairments of ocular origin from those originated in the brain. Future studies may also elucidate whether the ocular deficits are in part resulted from the retrograde effects of brain lesion caused by HIE, and vice versa.

Eyelid suture at P15 in the MD rats results in low-contrast vision of the same eye within a time-sensitive window critical for visual development^52^. Such deteriorated vision can lead to long-term synaptic depression along with reduced cell densities in the retina^52–54^. In amblyopia, the loss of vision is thought to be driven by the lack of visual brain development independent of eye abnormalities^52^. However, recent findings indicate that vision impairment in unilateral amblyopia is associated with altered retinal microvasculature and bilateral optic nerve hypoplasia with relative microphthalmia^55^, which is more notable in eyes with poorer vision^56–59^. Such differential extents of binocular involvements may potentially explain the significant volume reduction observed in the deprived eye of MD rats as compared to the ND rats but not the non-deprived eye of MD rats. Taken together, our results suggest that, along with impaired cortical development, peripheral factors may also be responsible for the decreased visual function upon prolonged MD at young ages.

Upon unilateral sensory deafferentation and loss of visual input in neonatal ME, the developing retinal tissues in the surviving eye are subject to a substantial decrease in normally occurring apoptosis along the uncrossed visual pathway^60,61^. This can lead to altered retinal soma size, distribution, and spontaneous activity compared to normal postnatal development^62,63^. As the VH serves as a metabolite reservoir for the retina while the VH and lens compositions can alter along with changes in the concentration of selected metabolites in the retina^36,64^, it is possible that the decrease in retinal apoptosis and metabolic demand may concur with the reduced VH and lens volumetric growth observed in the surviving eyes of our ME rats^65^. Adults who lost one eye early in life may show residual visual deficits even after prolonged monocular status^66^, yet the underlying mechanisms remain unclear. In addition to neuroplasticity^60^, our data supports the need to look into the uninjured eye independent of the brain after unilateral injury to one eye^67^.

Some of the limitations of this study include size differences between human and rodent eye development, the absence of fovea in non-primate species, and the less developed ciliary muscle in rodents rendering them unable to change their lens shape to mimic human conditions^25^. However, rodent ocular systems have important similarities to humans such as growth patterns and conserved retinal cell organization^29,68^, rendering them good models for development under disease conditions. In this study, logistic functions are used over linear functions for modeling normal postnatal ocular growth in Group 1 given the better fitting in terms of Bayesian information criteria and R^2^ values for all ACPC, lens, and VH volumes with logistic growth curves. It is expected that more data points from P28 to P60 and beyond could further improve the reliability of curve fitting in the adult stages and could be a subject for future investigation. The use of Cr as a contrast agent is limited to experimental animal studies only due to its toxic effects from the production of reactive oxidative species that can damage DNA and affect the functioning of antioxidant enzymes^69^. While local Cr administration at low dosages had no observable behavioral changes^16^, cautions should be taken when discerning the interactions of Cr with pathology that might arise in neonatal models in the future. Lastly, the different timings between MD and ME procedures could have accounted, at least in part, for the differences in ACPC and lens volumes observed between the 2 groups. Whereas MD was performed at P15 to minimize extrinsic visual input in one eye after natural eyelid opening, ME was performed at P1 to eliminate all spontaneous intrinsic retinal activity in one eye after birth. Recent studies indicated that surgical eye removal before and after P10 in rodents may result in different amounts of uncrossed optic nerve fiber projections from the remaining eye to the ipsilateral colliculus in the adult stage^60,70^. Future studies may assess how unilateral sensory deafferentation after P10 may affect ocular development as compared to ME at P1 or MD at P15 in rats.

MRI has lower resolution than optical imaging but has the advantage of whole-globe assessment without the need of complicated optical corrections. Future studies may combine MRI with ultrasound and optical imaging^71,72^ to leverage the strength of each technique for complementary global and local ocular growth assessments in fine details^11,24^. Future studies are warranted that use chromium-enhanced MRI in conjunction with visible-light optical coherence tomography to evaluate metabolic retinal development^73^. Prospective multiparametric MRI studies can also be conducted to analyze ocular physiology^10,74,75–78^ and determine structure–function relationships^79–82^. While the parameters of the current curve fits appear comparable to an ex vivo study on cryosection without fixation^1^, immunohistochemistry can be incorporated after preclinical MRI to study the molecular mechanisms of ocular growth. Moreover, in utero MRI of eye development^83^ can be further implemented beyond T2-weighted imaging using silent pulse sequences^84^.

In summary, in vivo high-field MRI allows for non-invasive evaluation of normal and impaired postnatal ocular development before and after natural eyelid opening in rats. Logistic growth curves were derived from the ACPC, lens, and VH volumes at varying rates within the first 60 days of normal postnatal development, indicative of the unique growth processes in different portions of the eye. Chromium-enhanced MRI appeared effective in examining the developing retina with relevance to lipid metabolism. Neonatal HIE, MD, and ME resulted in reduced ocular volumes to different extents and provided evidence to the emerging problems of why some impaired visual outcomes cannot be predicted by neurological assessments alone. This suggested the need to look into the eye alongside with the brain in evaluating neonatal visual impairments.

## Methods

### Animal preparation

Forty-two Sprague–Dawley rats were divided into six groups. In Group 1, seven rats were untreated and their course of normal development (ND) from newborn to adulthood was monitored at postnatal day (P) 1, P5, P10, P15, P28, and P60. In Group 2, the left eyes of nine other ND rats were intravitreally injected with 1 μL of 30 mM potassium dichromate paramagnetic contrast agent (Sigma-Aldrich, Missouri, USA) at P1 (n = 3), P7 (n = 3), and P14 (n = 3). In this experiment, each rat was only injected once to minimize injuries from multiple intraocular injections. This resulted in three animals at each age. In Group 3, seven rats underwent ligation of the left common carotid artery at P7. They were then enclosed in a hypoxic environment with an 8:92 oxygen–to-nitrogen ratio for 2 h at 36–37 degrees Celsius to induce neonatal hypoxic-ischemic encephalopathy (HIE)^85^. MRI was performed at P60 and compared with the ND rats in Group 1 at the same age. In Group 4, the right eyes of six rats were closed at P15 by suture to limit light exposure and induce monocular deprivation (MD) until the end experimental time point at P45. Group 5 consisted of five monocularly enucleated (ME) rats that had their right eyes removed at P1 through an incision in the conjunctiva followed by sectioning of the extraocular muscles and the optic nerve under inhaled isoflurane anesthesia. The empty socket was filled with oxidized regenerated cellulose Surgicel (Johnson & Johnson, New Jersey, USA) after eye enucleation^86^. All surgical procedures in Groups 2–5 were performed in under 30 min at 2–3% inhaled isoflurane concentration. At P45, both Groups 4 (MD) and 5 (ME) were compared to Group 6 that included eight separate ND control rats. T2-weighted imaging was performed to Group 1 and Groups 3–6 to examine the ocular anatomy and volumetry, whereas T1-weighted imaging was performed to Group 2 one day after chromium contrast agent administration^17^ to examine the signal enhancement and size changes during retinal development. All experimental protocols were approved by the Committee on the Use of Live Animals in Teaching and Research at the University of Hong Kong. All methods were carried out in accordance with the Association of Research for Vision and Ophthalmology Statement for the Use of Animals in Ophthalmic and Vision Research as well as the ARRIVE guidelines.

### MRI protocols

All MRI measurements were acquired using a 7-T Bruker scanner with a maximum gradient of 360 mT/m (70/16 PharmaScan, Bruker Biospin GmbH, Germany), a 72 mm birdcage transmit-only radiofrequency coil and an actively decoupled receive-only quadrature surface coil. Under inhaled isoflurane anaesthesia (3% induction and 1–1.5% maintenance), T2-weighted images were acquired with a spin-echo rapid-acquisition-with-relaxation-enhancement (RARE) pulse sequence covering the eye and the brain with repetition time (TR) = 4200 ms, echo time (TE) = 36 ms, RARE factor = 8, number of averages = 1, field of view (FOV) = 20 mm × 20 mm (for P1, P5, and P10) and 40 mm × 40 mm (for P15, P21, P28, and P60), in-plane acquisition resolution = 78 μm × 78 μm (for P1, P5, and P10) and 156 μm × 156 μm (for P15, P21, P28, and P60), and slice thickness = 0.8 mm. Chromium-enhanced MRI was performed using a T1-weighted RARE sequence with TR/TE = 475/8.8 ms, RARE factor = 4, number of averages = 26, FOV = 32 mm × 32 mm, in-plane acquisition resolution = 125 μm × 125 μm, and slice thickness = 0.8 mm.

### Data analysis

The anatomy of ocular structures was first examined qualitatively for each group using ImageJ version 1.52a (Rasband, W.S., ImageJ, U. S. National Institutes of Health, Bethesda, Maryland, USA, https://imagej.nih.gov/ij/, 1997–2018). In addition, volume rendering was performed to the eyes and brains of animals in Groups 1 and 3 using AMIRA software (Thermo Fisher Scientific, Waltham, Massachusetts, USA) to examine the general morphology over the course of normal development from newborn to adulthood or under HIE. Quantitatively, the volumes of the anterior chamber and posterior chamber (ACPC), lens, and vitreous humor (VH) in Groups 1 and 3–6 were segmented manually and measured using ImageJ. Subsequently, the measured values were compared within and between groups using analysis of variance (ANOVA) followed by Sidak multiple comparisons tests by GraphPad Prism version 8.1.2. (La Jolla, California, USA) (https://www.graphpad.com/). Since the ME rats in Group 5 had only one viable eye, only the non-enucleated eye was considered for this group. For Group 1, each volume was also plotted against postnatal age and regressed nonlinearly to produce a logistic growth curve fit with the equation: Y = Y_M_ * Y_0_/[(Y_M_ − Y_0_) * exp(− k * X) + Y_0_], where Y is volume in mm^3^, X is postnatal days, Y_0_ is the volume at birth in mm^3^, Y_M_ is the maximum volume in mm^3^, and k is the rate constant in inverse unit of X (day^−1^). For Group 2, the T1-weighted intensity and size of the retina in each eye were measured manually using ImageJ at the center of the eye. To ensure accurate regions-of-interest delineation, we increased the image contrast display in T1-weigthed imaging, and also took into account the higher contrast in the T2-weighted localizers of the same animals when defining the retina in the contralateral eye to the contrast agent injection. To account for potential systematic fluctuations between experimental sessions, the signal intensities in the retina of both eyes were normalized to their surrounding muscles before quantitative comparisons (Fig. 4a). These values were compared between contralateral eyes and across ages using ANOVA followed by Sidak multiple comparisons tests by GraphPad Prism. Results were considered significant when p < 0.05.
